# Responses of Soil CO_2_ Fluxes to Short-Term Experimental Warming in Alpine Steppe Ecosystem, Northern Tibet

**DOI:** 10.1371/journal.pone.0059054

**Published:** 2013-03-11

**Authors:** Xuyang Lu, Jihui Fan, Yan Yan, Xiaodan Wang

**Affiliations:** 1 Key Laboratory of Mountain Surface Processes and Ecological Regulation, Institute of Mountain Hazards and Environment, Chinese Academy of Sciences, Chengdu, Sichuan, China; 2 Xainza Alpine Steppe and Wetland Ecosystem Observation and Experiment Station, Institute of Mountain Hazards and Environment, Chinese Academy of Sciences, Xainza, Tibet, China; Lakehead University, Canada

## Abstract

Soil carbon dioxide (CO_2_) emission is one of the largest fluxes in the global carbon cycle. Therefore small changes in the size of this flux can have a large effect on atmospheric CO_2_ concentrations and potentially constitute a powerful positive feedback to the climate system. Soil CO_2_ fluxes in the alpine steppe ecosystem of Northern Tibet and their responses to short-term experimental warming were investigated during the growing season in 2011. The results showed that the total soil CO_2_ emission fluxes during the entire growing season were 55.82 and 104.31 g C m^-2^ for the control and warming plots, respectively. Thus, the soil CO_2_ emission fluxes increased 86.86% with the air temperature increasing 3.74°C. Moreover, the temperature sensitivity coefficient (*Q*_10_) of the control and warming plots were 2.10 and 1.41, respectively. The soil temperature and soil moisture could partially explain the temporal variations of soil CO_2_ fluxes. The relationship between the temporal variation of soil CO_2_ fluxes and the soil temperature can be described by exponential equation. These results suggest that warming significantly promoted soil CO_2_ emission in the alpine steppe ecosystem of Northern Tibet and indicate that this alpine ecosystem is very vulnerable to climate change. In addition, soil temperature and soil moisture are the key factors that controls soil organic matter decomposition and soil CO_2_ emission, but temperature sensitivity significantly decreases due to the rise in temperature.

## Introduction

Soil is the largest carbon pool in terrestrial ecosystems and contains more than 1500 Pg C, total carbon content in the soils of the whole planet is about three times larger than the total carbon present in terrestrial vegetation [1]. Soil carbon is returned to the atmosphere through the process of soil respiration, which refers to the total soil carbon dioxide (CO_2_) efflux at the soil surface, including autotrophic root respiration, and heterotrophic respiration associated with the decomposition of root-derived carbon, root and leaf litter, and soil organic matter [2]. Therefore, the CO_2_ flux from soil is a sensitive indicator of a physiological process in plant roots, soil microorganisms, or both [3]. Due to the magnitude of soil carbon pool, soils have the potential to influence atmospheric CO_2_ concentration. On a global scale, the CO_2_ flux from soils has been estimated to be on the order of 50–75 Gt C year^-1^ [4], [5] and about 11-fold greater than the fossil fuel combustion flux [6]. Thus, small changes in the size of this flux can have a large effect on atmospheric CO_2_ concentrations and potentially constitute a powerful positive feedback to the climate system [7].

Soil CO_2_ flux is affected in a complex way by temperature, moisture, soil properties, root exudation, and the quality and quantity of decomposing organic substrates [6], [8]. On a global scale, soil CO_2_ flux strongly correlates with annual mean temperature [5]. Numerous studies have shown that the soil CO_2_ emission rate increases exponentially or linearly with increasing temperature, with a temperature coefficient (*Q*_10_) of around 2.4 in temperate regions and of 2–8.8 in arctic and alpine regions [9], [10]. Global climate has experienced drastic changes in the 20^th^ century, and even more drastic changes are expected to take place in the 21^st^ century, which means that global temperature is projected to increase between 1.1 and 6.4°C by the year 2100 [11]. Global warming is predicted to increase the CO_2_ efflux from soil [12]. If an increased soil CO_2_ efflux is not balanced by an increased carbon uptake by vegetation photosynthesis, then warming can also turn ecosystems from carbon sinks into carbon sources [13], [14].

Soil moisture is another major factor that may influence soil CO_2_ emission in different ways. From laboratory studies and from theory, high water content can impede the diffusion of O_2_ in soil which constrains root respiration and organic matter decomposition. On the other hand, low soil water content can inhibit soil microbial activity and root activity [15]. The optimum soil moisture is usually somewhere near field capacity, when macropore spaces are mostly air-filled, O_2_ diffusion is facilitated, and when micropore spaces are mostly water-filled, soluble substrate diffusion is mostly facilitated [15]. And the threshold value of 20% volumetric water content over a depth of 0–10 cm is the low limiting value of soil moisture for soil respiration [16], [17]. The relationship between soil CO_2_ flux and soil temperature is modulated by soil moisture. The *Q*_10_ values decrease with decreasing moisture content when soil water content is lower than its optimum value [18], but an opposite trend is shown when soil retains water at contents higher than the optimum water content [19]. Future climate change maybe result in the alteration of annual amounts of precipitation and also the alteration of rain distribution, which may alter CO_2_ fluxes from soils, especially in semiarid and dry ecosystems where soil processes are water-limited [15], [19].

Soil CO_2_ flux is known to be highly variable, and its temporal variations have been described at various time scales, from diurnal to interannual variations. The seasonal variability is mostly explained by soil temperature and soil water content. Meanwhile, some short-term temporal variability could be explained by litter moisture, rain events, soil rewetting after a drought period and other environmental factors [20]. The *Q*_10_ function is considered a good choice for estimating the total annual soil CO_2_ flux because it integrates all the processes that may influence diurnal, seasonal and annual soil CO_2_ emissions [10], [11]. However, the *Q*_10_ of soil respiration has a large temporal variation, and that the use of a constant *Q*_10_ may result in significant errors in predicting future soil carbon losses. Thus, analysis at a seasonal or finer temporal resolution is urgently needed to improve our understanding of the interactions between environmental variables and soil CO_2_ emissions, and to help reduce the uncertainty about the temperature dependence of soil CO_2_ flux [21], [22].

Alpine regions are critical for studies of global change and monitors of ecological changes because they are sensitive and fragile ecosystems and are among the most extreme terrestrial environments on Earth [23], [24]. In addition, alpine regions are also believed to be exposed to a rate of warming higher than the global mean warming level [25]. The Northern Tibet region, located in the interior of the Tibetan Plateau, is more than 4,500 m above sea level and has peaks more than 6 km high. This region is the headwater of many high mountain lakes and important rivers in China as well as other Asian countries, such as the Yangtze River, Nu (the Salween River), and Lancang (the Mekong River) [26], [27]. Alpine grassland is the dominant ecosystem in this region, occupying about 94% of total area. It is not only the most important and largest ecosystem in the area, but also a key resource supporting local people’s subsistence [28]. Owing to its extremely harsh natural environment and average elevation of over 4,500 m, the alpine grassland of Northern Tibet is a fragile ecosystem that is sensitive to climate change and human activities [26], [27].

In the present study, we increased the temperature of the alpine steppe ecosystem in Northern Tibet for four months. We investigated how experimental warming affected the soil CO_2_ fluxes in this alpine steppe grassland ecosystem. Specifically, we hypothesized that: (1) an increase in temperature would stimulate soil CO_2_ fluxes at different timescales (i.e., daily, monthly and seasonally) during the growing seasons. This is because low temperature is a limiting factor for ecological processes in high-altitude ecosystems. Therefore, soil respiration is predicted to increase with increasing soil temperature; (2) soil environmental factors, including soil temperature and moisture were key factors that influence soil CO_2_ fluxes in this alpine steppe region; and (3) the temperature coefficient (*Q*_10_) of alpine steppe soil CO_2_ fluxes would decrease because of experimental warming.

## Materials and Methods

### Site description

Studies were conducted in permanent plots at the Xainza Alpine Steppe and Wetland Ecosystem Observation and Experiment Station (30°57′N, 88°42′E, 4675 m a.s.l) located in Xainza County, Northern Tibet, China. This area is located in a cold and semi-arid plateau monsoon climate region. According to 30-year records from the meteorological station (4671 m a.s.l.) located about 2 kilometers away from the study site, the annual mean air temperature was 0°C, the mean air temperature during January was –10.1°C, and the mean air temperature during July was 9.6°C. There is no absolute frost-free season. The annual period of direct solar radiation reaching the earth surface is 2916 hours. The average annual precipitation is 300 mm, most of which occurs during May-September period. The natural environment of this area is extremely harsh and belongs to a region of seasonally frozen soil which is generally quite poor in nutrients. The soil bulk density was 1.76 g·cm^-3^ with pH 8.78. The soil organic C and total N, total P, total K contents of the soil were 11.12, 1.03, 0.52, 31.22 g·kg^-1^, respectively. And 0.25–0.05 mm and 0.5–0.25 mm predominated in the soil particle fraction. The selective alpine steppe had less than 20% vegetation coverage, with forage grasses *Stipa purpurea* and *Carex moorcrofti* as the dominant species and *Oxytropis*. spp., *Artemisia capillaris* Thunb., *Aster tataricus* L. as the companion species. In addition, no specific permits were required for the described field studies and the field studies did not involve endangered or protected species.

### Experimental design and microclimate monitoring

Three open top chambers (OTCs) were randomly set up in the alpine steppe permanent plots to increase air and soil temperature. One control plot was established randomly in the vicinity of each OTC. The distance between each OTC was roughly 20 m, which ensured that all of the plots had similar slopes and aspects. The OTCs used in this study were hexagonal and 160 cm high, made of solar transmitting material, with 2.60 m^2^ at the ground area tapering to 0.94 m^2^ at the open-top area. All the selected plots were expected to be similar in microhabitat characteristics. The OTC installations were completed in October 2010 and observations were initiated from May 2011.

In order to quantify the environmental factors affected by the OTCs, the automatic climate recording systems were set up in the control and warming plots. Air temperatures at 35 cm above the soil surface were measured in the center of each plot by using humidty/temp sensor with radiation shield (Decagon, Washington, DC, USA). Soil temperature and soil moisture at depths of 10 cm were measured through 5TM soil temperature and moisture sensors (Decagon, Washington, DC, USA). Soil temperature and moisture measurements were taken at 10 cm soil depth because most roots and organic matter are found in the upper 10 cm of the soil. The measurements of soil temperature and moisture were carried out in the area of the OTCs without rainfall interception to avoid any edge effects of the OTCs. Data were taken at 60 -min intervals from early May to late September 2011 and were stored on EM50 digital/analog data logger (Decagon, Washington, DC, USA).

### Soil CO_2_ flux measurement

Soil CO_2_ fluxes were measured by using the Li-8100A Automated Soil CO_2_ Flux System (Li-Cor Inc., Lincoln, NE, USA). To measure soil CO_2_ flux, the chambers (20 cm in diameter and 5 cm in height) were inserted into the soil in each plot in early May 2011. All living plants inside the soil collars were removed by hand at least one day prior to the measurements to exclude plant respiration from the aboveground parts and measurements of soil CO_2_ fluxes were also taken in the center of the plot to avoid edge effects. During the growing season of 2011, the soil CO_2_ fluxes were measured every 4–6 days depending on weather conditions. For a consistent measurement protocol, the soil CO_2_ fluxes between 08:30 and 11:30 a.m. on clear days represent a one-day average flux according to the diurnal gas flux variation measurement. The order of CO_2_ flux measurements was random, but a measurement in a control plot was always followed by a measurement in the adjacent warming plot. Soil CO_2_ flux in each chamber was measured continuously for three cycles, and the three measurements were averaged to produce a mean soil flux. In addition, soil CO_2_ fluxes were also measured at 2–hour intervals from 08:00 to 20:00 local time with twice or thrice a month to capture the diurnal variation pattern.

### Statistical analysis

The total amount of soil CO_2_ emission during the growing season of 2011 was estimated by linear interpolation among the sequential soil CO_2_ emission rates measurements in our sampling date time series (MATLAB, Curve Fitting Tool). To examine the temperature sensitivity of soil CO_2_ fluxes, nonlinear exponential regression models were conducted using *Y* = *a*e*^bT^*, where *Y* is the soil CO_2_ flux, *T* is the soil temperature, coefficient *a* is the intercept of the soil CO_2_ flux when temperature is zero, and coefficient *b* represents the temperature sensitivity of the soil CO_2_ flux. The temperature coefficient (*Q*_10_) was used to assess the temperature dependence of soil CO_2_ fluxes at each time the respiration rates were measured. According to the definition of *Q*_10_, the *Q*_10_ value from the equation (*Y* = *a*e*^bT^*) was calculated as: *Q*_10_ = *R_T_*_+10_/*R_T_*, where *R_T_* and *R_T_*_+10_ are the soil CO_2_ emission rates at temperatures *T* and *T*+10, respectively. The *Q*_10_ values were calculated for each of the control and warming treatments by using all of the data in the diurnal data set and in the seasonal data set, respectively. Simple correlation analyses were performed to test the possible dependency of the soil CO_2_ fluxes on soil moisture. And the stepwise regression procedures (SPSS Inc., USA) were used to quantitatively assess the effects of soil temperature interaction with moisture on the soil CO_2_ fluxes. For specific sampling dates, one-way ANOVA was used to compare the effect of the experimental warming and a Least Significant Difference (LSD) test was used to distinguish the difference at *p*  =  0.05. General linear model measures defined the factors (SPSS Inc., USA) with warming and sampling date as the main factors including their interactions, were applied to test the effects of the main factors on the seasonal variations of soil CO_2_ fluxes. Before analysis, all data were tested for the assumptions of ANOVA with the homogeneity of variance test (SPSS Inc., USA). If the data were heterogeneous, they were ln-transformed before analysis. All analyses were performed using the SPSS 11.5 statistical software package (SPSS Inc., USA).

## Results

### Microclimates

**Figure 1:**
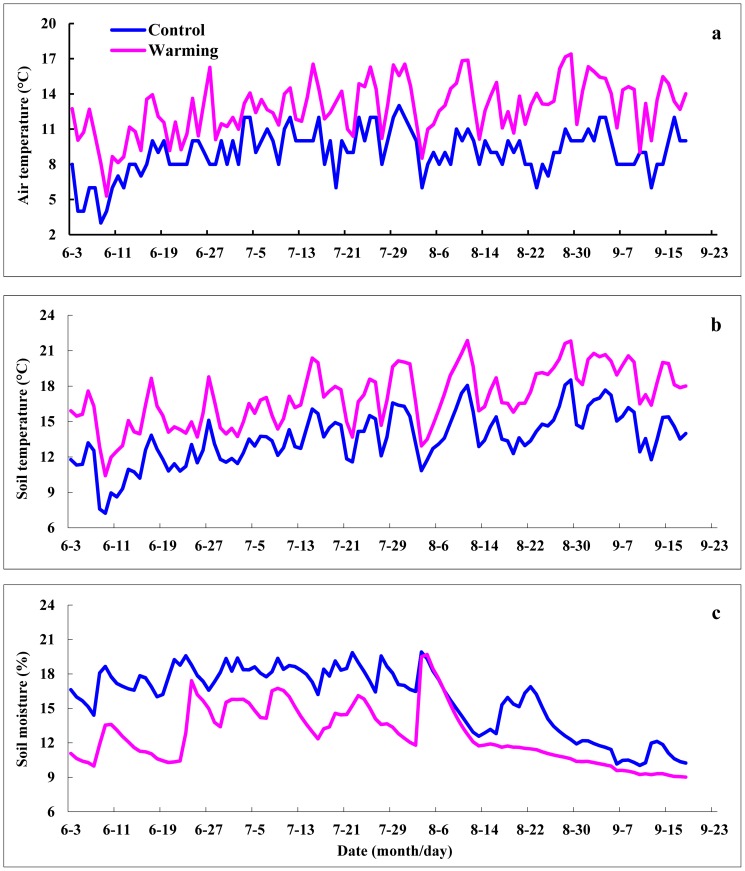
Microclimates in control and warming plots in an alpine steppe during the growing season. (a) Daily mean air temperature, (b) daily mean soil temperature and (c) daily mean soil moisture in the alpine steppe control and warming plots during the growing season.

The OTCs resulted in an increase of air and soil temperature in the experimental plots. Mean air temperature during sampling time (from 3rd June to 18th September) were 9.04°C and 12.78°C and mean soil temperature at 10 cm depth were 13.59°C and 17.05°C for the control and warming plots, respectively (Fig. 1a, 1b). These results indicate that in contrast to the control plots, the air and soil temperatures in the OTCs increased by an average of 3.74°C and 3.46°C, respectively, in the alpine steppe throughout the growing season of 2011. Conversely, soil moisture content at 10 cm depth declined by 3.19% because of warming. Mean soil moisture contents were 15.86% and 12.67% for the control and warming plots respectively (Fig. 1c). Microclimates were significantly different between the control and warming plots (air temperature: *p* < 0.001, soil temperature: *p* < 0.001, soil moisture: *p* < 0.001), but air and soil temperature, as well as soil moisture content between the control plots and the warming plots exhibited similar seasonal patterns during the growing season.

### Diurnal variation of soil CO_2_ fluxes

**Figure 2:**
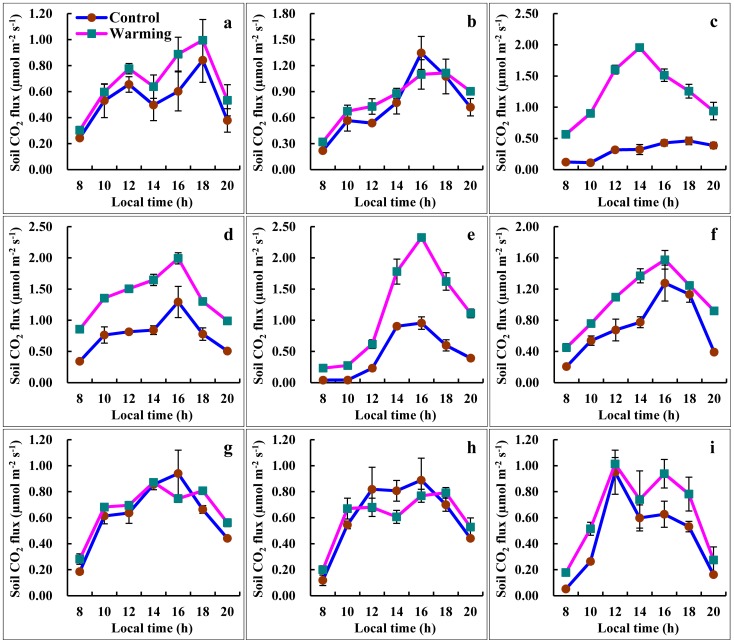
Daily variation of soil CO_2_ fluxes on nine representative days. (a) 5th June, (b) 16th June, (c) 9th July, (d) 24th July, (e) 4th August, (f) 15th August, (g) 24th August, (h) 1st September and (i) 20th September in the alpine steppe control and warming plots during the growing season. Each data point represents the mean of nine replicates, and error bars indicate ± SE.

The diurnal variation patterns of soil CO_2_ fluxes of the alpine steppe during the growing season of 2011 are shown in Figure 2. In the nine measurement days, the soil CO_2_ fluxes increased from 08:00, reached maximum mainly between 12:00 and 16:00, and subsequently gradually decreased at both the control and warming plots. Compared with the control plots, warming promoted the soil CO_2_ release of the alpine steppe, and the soil CO_2_ fluxes of warming plots were higher than those of the control plots in all measurement days. Especially in 9th July (Fig. 2c), 24th July (Fig. 2d) and 4th August (Fig. 2e), the differences of soil CO_2_ fluxes between the control plots and the warming plots were statistically significant (9th July: *p* < 0.001, 24th July: *p*  =  0.006, 4th August: *p*  =  0.048). For instance, the maximum of mean diurnal soil CO_2_ fluxes obtained in 24th July in both the control and warming plots, were 0.76 and 1.38 µmol m^-2^ s^-1^, respectively. The mean diurnal soil CO_2_ fluxes of the warming plots were about twice higher than those of the control plots.

The diurnal patterns observed in our study were correlated with the diurnal variation in soil temperature. Exponential equations can generally describe the relationship between the diurnal variation of soil CO_2_ fluxes and the soil temperature at 10 cm depth (Table 1), the determination coefficients (*r* ^2^) were 0.28 (control plots: *p* < 0.001) and 0.11 (warming plots: *p*  =  0.009), respectively. The temperature coefficients (*Q*_10_) of the control and warming plots which calculated from the regression slope of the diurnal variations of soil CO_2_ fluxes were 2.10 and 1.41, respectively. That is to say, the *Q*_10_ decreased by about 32.86% due to warming treatment. The diurnal variation of soil CO_2_ fluxes was not significantly correlated with soil moisture in the control plots, but increased significantly with increasing soil moisture in the warming plots (*r*  =  0.56, *p* < 0.001).

**Table 1:** The regression analyses results for soil CO_2_ fluxes diurnal variation and seasonal variation.

Soil CO_2_ Fluxes	Soil factor	Plots	Regression equation	*r* ^2^	*p*	*Q*_10_
Diurnal variation	Soil temperature	Control	*Y* = 0.1502e^0.0743*T*^	0.28	<0.001	2.10
		Warming	*Y* = 0.4299e^0.0336*T*^	0.11	0.009	1.41
	Soil moisture	Control	Not pass *F* test	–	–	–
		Warming	*Y* = 0.0403*M*+0.5119	0.31	<0.001	–
Seasonal variation	Soil temperature	Control	Not pass F test	–	–	–
		Warming	*Y* = 0.3792e^0.0591*T*^	0.14	0.017	1.81
	Soil moisture	Control	Not pass *F* test	–	–	–
		Warming	*Y* = 0.0310*M*+0.4965	0.37	<0.001	–

*Y*: Soil CO_2_ fluxes; *T*: Soil temperature; *M*: Soil moisture

### Seasonal variation of soil CO_2_ fluxes

**Figure 3:**
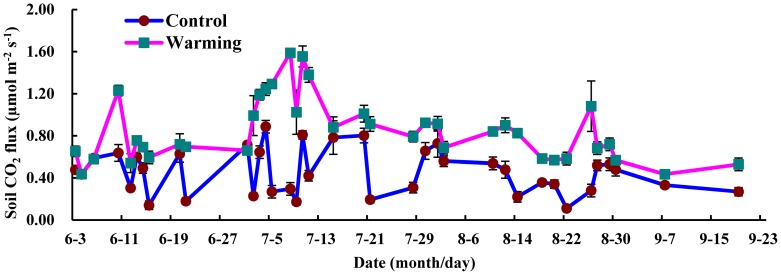
Seasonal variation of soil CO_2_ fluxes in the alpine steppe during the growing season. Symbols and data points are as in Figure 2.

**Figure 4:**
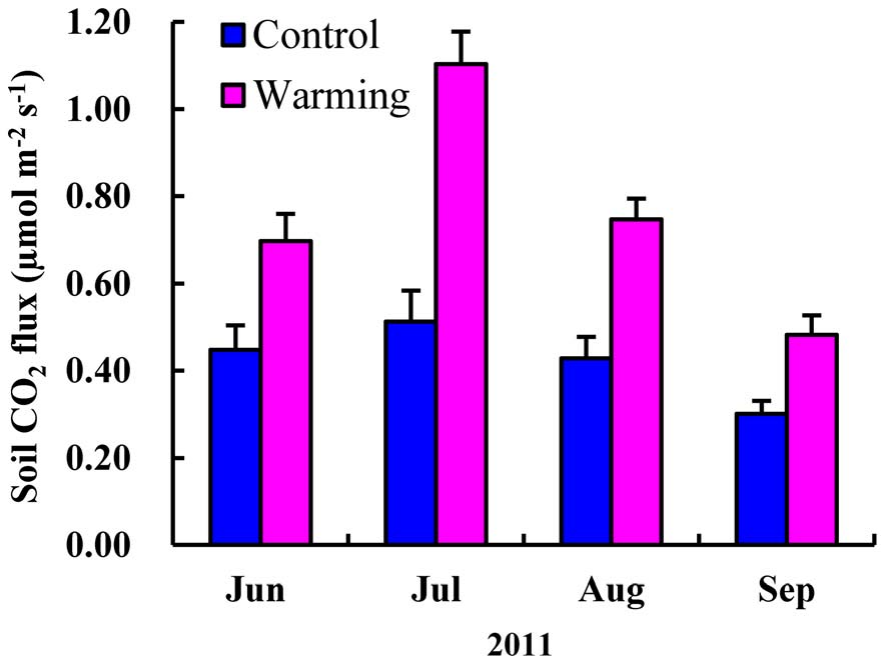
Monthly average values of soil CO_2_ fluxes in the alpine steppe during the growing season.

Soil CO_2_ fluxes showed seasonal variations ranging from 0.11µmol m^-2^ s^-1^ to 0.89 µmol m^-2^ s^-1^ in the control plots and from 0.44 µmol m^-2^ s^-1^ to 1.59 µmol m^-2^ s^-1^ in the warming plots throughout the growing season (Fig. 3). In general, the fluctuation ranges of soil CO_2_ flux were higher in July and August than in June and September. The monthly mean values of soil CO_2_ fluxes in both the control and warming plots increased from June, reached the maximum in July and subsequently decreased in August and September (Fig. 4). During the growing season of 2011, the total amount of soil CO_2_ emission from the alpine steppe control plots was 55.82 g C m^-2^. Warming markedly increased the soil CO_2_ fluxes over the growing season, across all measuring dates, and the average soil CO_2_ emission rate increased by 86.86%. The total amount of soil CO_2_ emission was 104.31 g C m^-2^ in the warming plots. Results from the statistical analyses demonstrate that warming, sampling time, and their interaction were all statistically significant as the effect for soil CO_2_ fluxes (warming: *F*_1_  =  181.32, *p* < 0.001; sampling date: *F*_39_  =  4.75, *p* < 0.001; warming × sampling date: *F*_39_  =  2.92, *p* < 0.001). In the control plots soil CO_2_ fluxes were not significantly correlated with soil temperature and soil moisture (Table 1), but in warming plots soil CO_2_ fluxes increased significantly with increasing soil temperature (*r*  =  0.37, *p*  =  0.017) with the *Q*_10_ was 1.81 which calculated from the regression slope of the seasonal variations of soil CO_2_ fluxes. In the warming plots soil CO_2_ fluxes also significantly correlated with soil moisture (*r*  =  0.61, *p* < 0.001).

## Discussion

### Warming effects

In this study, OTCs were used to determine the responses of soil CO_2_ fluxes to the artificial warming of the alpine steppe ecosystem in Northern Tibet. The OTC was the method of passive ecosystem warming studies which were used extensively from 1980 s [29], [30], [31]. Over the growing season, the OTCs increased the daily mean air and soil temperature by an approximate average of 3.74°C and 3.46°C (Fig. 1). The magnitude of soil warming in our study is a little higher than that seen in other studies [32], [33], possibly because of the strong solar radiation in the Tibetan Plateau. The soil moisture content at 10 cm depth of the control plots was 3.19% lower than that of the warming plots due to experimental warming. The OTCs elevate air and soil temperature, which may lead to a small decrease in soil moisture within the chambers by increasing ecosystem evapotranspiration [31], [34].

Climate warming in high latitude and high altitude is expected to strongly affect the carbon balance of tundra and alpine ecosystems, some studies even suggest that the carbon balance of these ecosystems is already changing [10], [33]. The Tibetan Plateau is experiencing climatic warming and the region is predicted to experience “much greater than average” increases in surface temperatures in the future [11]. The magnitude of short-term warming (3.74°C) in this study was close to the warming tendency (3.8°C) of the Tibetan Plateau by the end of the 21^st^ century which projected by the Intergovernmental Panel on Climate Change (IPCC) in A1B climate scenario [11]. The Tibetan Plateau is also one of the most sensitive areas to global climate change [10]. Experimental warming resulted in an approximately 87% increase in the total amount of soil CO_2_ emission in the alpine steppe during the growing season of 2011 which supported our hypothesis that an increase in temperature will stimulate soil CO_2_ fluxes. Several other studies also demonstrated that warming obviously stimulated soil or ecosystem respiration in the Tibetan Plateau. For instance, Xu et al [35] found that warming increased the average soil CO_2_ efflux by 10.6% in the plantation and by 15.4% in the natural forest at the Miyaluo experimental forest of Lixian county, eastern Tibetan Plateau. Lin et al [10] found that warming significantly increased the seasonal average soil respiration by 9.2%, which mainly occurred early in the growing season at the Haibei alpine meadow ecosystem research station, northeastern Tibetan Plateau. However, the increase in soil respiration of more than 80% in this study is much higher than the effect size reported elsewhere, even on the Tibetan Plateau. Probably because that the soil CO_2_ flux was considerable low (0.47 µmol m^-2^ s^-1^) under natural conditions (the control plots) in this alpine steppe, warming could promote soil CO_2_ emissions easily and formed a pulse response in the short term. However, the emission rate was still considerable low (0.84 µmol m^-2^ s^-1^) under warming conditions (the warming plots) in contrast to those of other ecosystems [10], [35]. Numerous studies reported that elevated temperatures increased soil CO_2_ flux because warming increased soil and litter decomposition [12], [36]. Nevertheless, how experimental warming affects the soil CO_2_ flux of the alpine steppe still remains to be clarified because the root respiration and soil microbial respiration were not distinguished in the present study. Thus, more detailed studies regarding the partitioning of soil respiration into root and microbial respiration, and detailed physiological responses of these components covering prolonged observation periods for the responses to warming should be conducted to further elucidate the underlying mechanisms.

### Soil environmental factors

Although various environmental factors affect the biological and physical processes controlling soil CO_2_ emission, soil temperature and moisture are the most important factors controlling soil CO_2_ fluxes [37], [38]. The temporal variations of soil CO_2_ fluxes were greater in both natural and warming conditions (Fig. 3), which seems to match the higher variability in air temperature and moisture in this alpine region (Fig. 1). In the alpine steppe ecosystem, the diurnal variations of soil CO_2_ flux were significantly correlated with soil temperature at 10 cm depth in both the control and warming plots (Table 1). The seasonal variations of soil CO_2_ fluxes were not significantly correlated with soil temperature in the control plots, but increased significantly with increasing soil temperature in the warming plots (Table 1). This finding is generally in agreement with previous reports for tundra and alpine ecosystems, in which soil temperature was the important factor that affect soil CO_2_ emission [33], [34]. However, the determination coefficients (*r* ^2^) were considerable low in the present study, only 28% (control plots) and 11% (warming plots) of the diurnal variations of soil CO_2_ flux, 14% (warming plots) of the seasonal variations of soil CO_2_ flux were explained by soil temperature. These low values maybe because that the background soil temperatures were considerable low in alpine grassland ecosystems at all times. Although an increasing trend in the warming stimulation of soil CO_2_ fluxes was observed, that fluxes were still universally limited due to low soil temperatures. Other biotic and abiotic factors, such as clipping, which has been demonstrated probably causing an increase of both soil and root respiration due to an increase in soil temperature on the clipped plots, and belowground biomass, may account for more variations of soil CO_2_ flux in this alpine grassland [39], [40].

The temperature coefficient (*Q*_10_), which refers to the factor by which soil CO_2_ flux increases with an increase in temperature of 10°C, is considered one of the most important parameters used to assess the temperature sensitivity of soil respiration [38]. The *Q*_10_ of the alpine steppe was 2.10 in the control plots and 1.41 in the warming plots, which calculated from the regression slope of the diurnal data set, were close to the range reported by previous studies in the alpine region [10], [41]. The hypothesis was that *Q*_10_ of the alpine steppe soil CO_2_ fluxes will decrease because of experimental warming. This hypothesis was supported because experimental warming resulted in the *Q*_10_ decreased 0.69 in the alpine steppe ecosystem. Experimental warming resulted in an approximately 87% increase in soil CO_2_ emission and 0.69 reduction in *Q*_10_ during the growing season. It suggests that the alpine steppe ecosystem in Northern Tibet is very vulnerable to climate change, at least in the short term. However, the decrease in *Q*_10_ indicates that this pulse response may be short lived because soil respiration was so quick to acclimatize to warmer temperatures. After a few months of elevated temperatures, this alpine steppe soils will probably acclimatize gradually to the new temperature regime with the decreasing in *Q*_10_. This decrease in temperature sensitivity of soil CO_2_ flux under warming could result from several mechanisms, including concurrent reduction in plant production leading to less root respiration, soil drying reducing root and microbial activity, and substrate limitation [42], [43]. However, to support these hypotheses it would be necessary to determine the plant aboveground and belowground live biomass, soil carbon transformation microorganisms and enzyme activities, substrate quality and quantity in future studies.

Soil moisture is another important factor influencing soil respiration. Soil CO_2_ flux is low in dry conditions and increases to a maximum at intermediate moisture levels until it begins to decrease when moisture content excludes oxygen [44], [45]. On the regional scale, soil moisture together with belowground biomass, rather than soil temperature accounted for the majority (82%) of spatial patterns of alpine grassland soil CO_2_ flux in the Tibetan Plateau [39]. In the present study, the soil CO_2_ diurnal fluxes of the warming plots were significantly higher than those of the control plots on 9th July, 24th July and 4th August (Fig. 2). Comparison the soil moisture of the measurement nine days, the soil moisture of these three days exceeded or approached 20% but the soil moisture of other six days were far less than 20%, which 20% soil moisture at a depth of 0–10 cm was thought as the soil moisture threshold value for soil respiration [16], [17]. Thus, perhaps under no soil moisture limiting conditions, warming promoted soil released more CO_2_ to atmosphere. Both the diurnal and seasonal variations of soil CO_2_ fluxes were not significantly correlated with soil moisture in the control plots. However, after the experimental warming due to OTCs, the diurnal and seasonal variations of soil CO_2_ fluxes increased significantly with increasing soil moisture (Table 1). A possible reason for this increase is that, in natural conditions, soil temperature is the primary key factor that influences root respiration and soil microbial respiration processes. However, experimental warming resulted in a 3.19% decline in soil moisture of the warming plots compared with that of the control plots. Maybe it leads to soil moisture also becomes the key factor that controls the soil respiration processes. If the soil moisture function was applied to the residuals of the soil temperature nonlinear exponential regression model, the addition of soil moisture function to the soil temperature-only model significantly increased the predictive power of the warming plots in both the diurnal variations (*r* ^2^  =  0.11 for soil temperature, *r* ^2^  =  0.49 for soil temperature + soil moisture) and the seasonal variations (*r* ^2^  =  0.14 for soil temperature, *r* ^2^  =  0.51 for soil temperature + soil moisture). Similar empirical models which enhanced the predictive power of the variation in soil CO_2_ emission rates by utilizing both soil temperature and soil moisture have also been reported in the uplands and wetlands of other regions [46], [47].

## Conclusion

Three open top chambers (OTCs) were set up in the alpine steppe of Northern Tibet to investigate soil CO_2_ fluxes responses to short-term experimental warming. The OTCs increased the daily mean air temperature by an approximate average of 3.74°C during the growing season of 2011 which was close to the warming tendency (3.8°C) projected by the IPCC in A1B climate scenario on the Tibetan Plateau by the end of 21^st^ century [11]. Experimental warming resulted in an approximately 87% increase in soil CO_2_ emissions and a 0.69 reduction in *Q*_10_ in this alpine steppe ecosystem, which indicate that this alpine ecosystem is very vulnerable to climate change. The increasing carbon losses under warming may be compensated by increasing the net primary productivity of vegetation. Thus, more detailed studies regarding ecosystem-level carbon exchanges, such as vegetation photosynthetic carbon fixation, and plant respiration, are necessary to further elucidate the processes and underlying mechanisms of the carbon budget of alpine steppe ecosystem under climate warming. Based on the present study, the soil temperature and soil moisture could partially explain the temporal variations of soil CO_2_ fluxes. Nevertheless, what are the crucial factors which regulate the soil CO_2_ emissions in alpine steppe ecosystem under natural and warming conditions still remain to be clarified, it would be necessary for future research to distinguish root respiration and soil microbial respiration as well as determine more relevant biotic and abiotic factors.
